# AI-assisted biparametric MRI surveillance of prostate cancer: feasibility study

**DOI:** 10.1007/s00330-022-09032-7

**Published:** 2022-08-12

**Authors:** C. Roest, T.C. Kwee, A. Saha, J.J. Fütterer, D. Yakar, H. Huisman

**Affiliations:** 1 Department of Radiology, University Medical Center Groningen, Kochstraat 250, 9728 KL Groningen, the Netherlands; 2 Department of Medical Imaging, Radboud University Nijmegen Medical Centre, Geert Grooteplein Zuid 10, 6500 HB Nijmegen, the Netherlands

**Keywords:** Prostatic neoplasms, Magnetic resonance imaging, Deep learning

## Abstract

**Objectives:**

To evaluate the feasibility of automatic longitudinal analysis of consecutive biparametric MRI (bpMRI) scans to detect clinically significant (cs) prostate cancer (PCa).

**Methods:**

This retrospective study included a multi-center dataset of 1513 patients who underwent bpMRI (T2 + DWI) between 2014 and 2020, of whom 73 patients underwent at least two consecutive bpMRI scans and repeat biopsies. A deep learning PCa detection model was developed to produce a heatmap of all PIRADS ≥ 2 lesions across prior and current studies. The heatmaps for each patient’s prior and current examination were used to extract differential volumetric and likelihood features reflecting explainable changes between examinations. A machine learning classifier was trained to predict from these features csPCa (ISUP > 1) at the current examination according to biopsy. A classifier trained on the current study only was developed for comparison. An extended classifier was developed to incorporate clinical parameters (PSA, PSA density, and age). The cross-validated diagnostic accuracies were compared using ROC analysis. The diagnostic performance of the best model was compared to the radiologist scores.

**Results:**

The model including prior and current study (AUC 0.81, CI: 0.69, 0.91) resulted in a higher (*p* = 0.04) diagnostic accuracy than the current only model (AUC 0.73, CI: 0.61, 0.84). Adding clinical variables further improved diagnostic performance (AUC 0.86, CI: 0.77, 0.93). The diagnostic performance of the surveillance AI model was significantly better (*p* = 0.02) than of radiologists (AUC 0.69, CI: 0.54, 0.81).

**Conclusions:**

Our proposed AI-assisted surveillance of prostate MRI can pick up explainable, diagnostically relevant changes with promising diagnostic accuracy.

**Key Points:**

*• Sequential prostate MRI scans can be automatically evaluated using a hybrid deep learning and machine learning approach.*

*• The diagnostic accuracy of our csPCa detection AI model improved by including clinical parameters.*

**Supplementary Information:**

The online version contains supplementary material available at 10.1007/s00330-022-09032-7.

## Introduction

Prostate cancer (PCa) is the second most common malignancy among men worldwide, making up over one-fifth of cancer diagnoses in men [1]. In 2020, there were an estimated 1.4 million new cases and 375,000 PCa-related deaths worldwide. The introduction of prostate-specific antigen (PSA) screening for the detection of PCa has led to an overdiagnosis of low-risk PCa. For men with localized, low-risk PCa, curative treatment has limited benefit and is associated with a reduced quality of life [2, 3]. Active surveillance (AS) is a widely used management plan to reduce the overtreatment of low-risk PCa [4]. Men on AS are closely monitored using PSA measurements and repeat biopsies, to detect disease progression early and to maximize the efficacy of subsequent curative treatment.

Serial MRI provides a noninvasive method for monitoring patients with PCa and is routinely used by radiologists to compare the findings between different patient visits and to report on radiological stability or progression [5]. The PRECISE guidelines [6] are the current best practice for reporting serial MRI findings in the AS setting, based on changes in size and characteristics of PCa lesions. Several studies reported a promising diagnostic accuracy for MRI in AS of low-risk PCa [7, 8]. However, reported diagnostic accuracies are currently still too low to support MRI-based surveillance as a reliable alternative for repeat biopsies in AS protocols [9].

MRI surveillance may be improved with artificial intelligence (AI). Numerous studies have applied deep learning (DL) for the automatic detection and characterization of PCa on MRI at a single time point [10, 11]. However, none has thus far explored the use of differential information on sequential prostate MRI examinations. The straightforward development of AI for surveillance is challenging, due to a general lack of large numbers of sequential MRI examinations. We propose a data-efficient hybrid machine learning algorithm. It leverages previous research in AI where a large volume of single–time point MRI studies can generate interpretable features. The proposed system is trained such that it detects and tracks all PCa lesions, but discriminates clinically significant (cs) from non-significant PCa by using static and dynamic PCa features.

This study aims to assess the feasibility of AI-assisted biparametric MRI (bpMRI) surveillance of the prostate for the automatic detection of csPCa. We will (1) compare a single–time point bpMRI scan surveillance AI versus a multi–time point bpMRI scan surveillance AI; (2) investigate if the inclusion of clinical parameters results in a further improvement; and (3) investigate how the performance of the best AI surveillance model compares to the performance of an experienced radiologist.

## Materials and methods

### Dataset and preprocessing

This study uses a multi-center database collected from two tertiary care academic institutions, and ten non-academic institutions (Supplementary Table 1). The institutional review board of both tertiary care academic institutions approved this study and waived the need for informed consent.

**Fig. 1:**
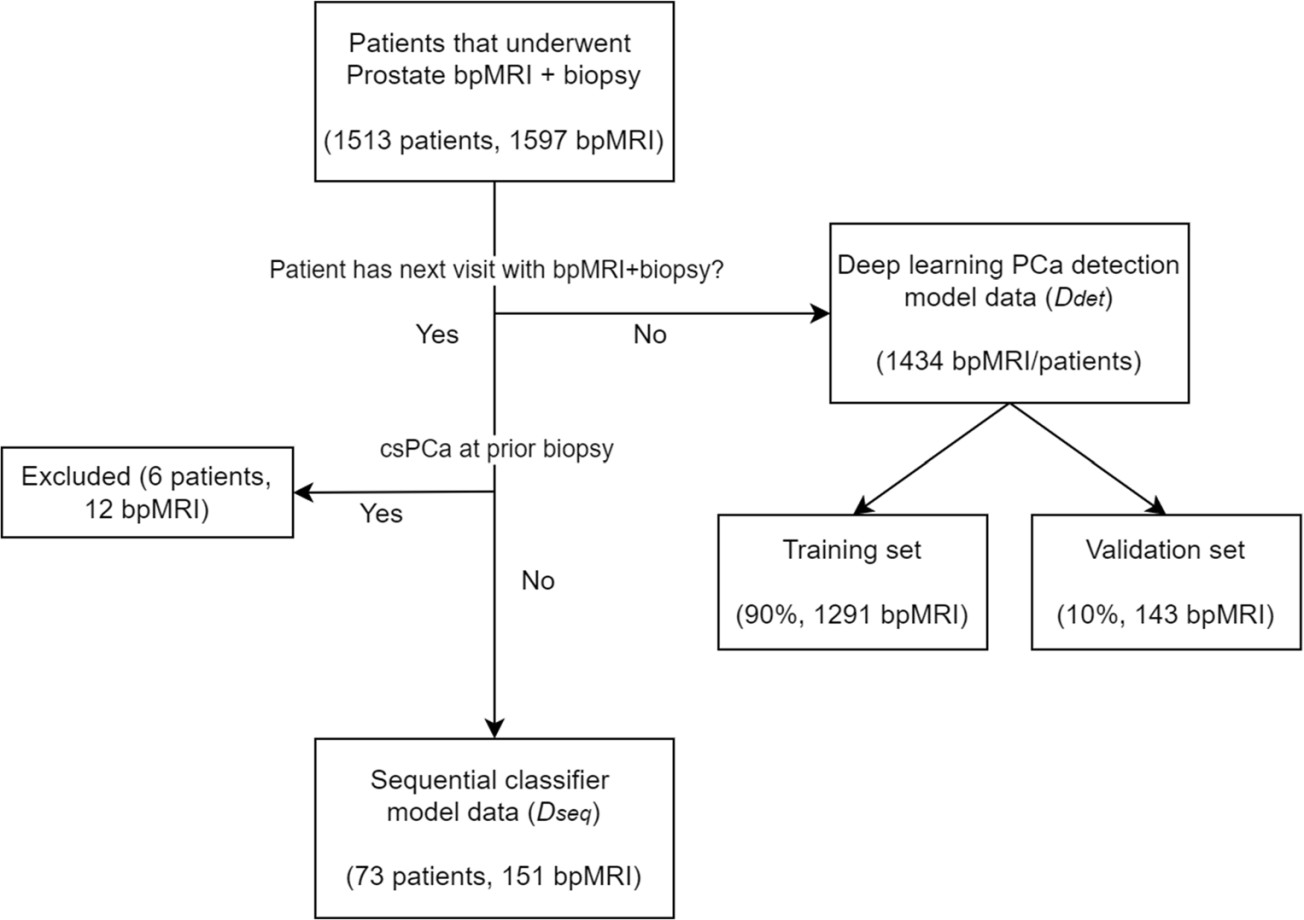
Overview of the division of the dataset

The data were divided into two cohorts: The first cohort consisted of bpMRI scans of patients who were scanned only once (*n* = 1434), which was used to train a DL model for the detection of PCa lesions. The second cohort consisted of serial bpMRI scans (prior + current) of patients who all had undergone a targeted or systematic biopsy at both examinations, without signs of csPCa at the prior biopsy (*n* = 73), which was used for training and evaluating the sequential classifier (Fig. 1). We refer to these datasets as *D*_det_ and *D*_seq_, respectively. Five patients were scanned three times, from whom two scan pairs were derived each (baseline ➔ follow-up 1, baseline ➔ follow-up 2), resulting in a total of 78 current examinations in *D*_det_.

Considering the lack of an undisputedly proven value of dynamic contrast-enhanced imaging in prostate MRI, we developed our system using bpMRI [12]. In accordance with the PIRADS guidelines, we used the transverse T2-weighted (T2W), apparent diffusion coefficient (ADC), and calculated high *b*-value (≥ 1400 s/mm^2^) diffusion-weighted imaging (DWI) sequences, which were routinely acquired in each patient [12]. The MRI studies adhered to the protocol recommendations of PIRADS guidelines; therefore, there were no major differences in the imaging between institutions. Ranges for MRI protocol parameters are provided in Supplementary Table 1. All images were prospectively evaluated for quality issues by an expert radiologist. All scans were resampled to an identical voxel spacing of 0.5 × 0.5 mm^2^ (slice thickness 3.6 mm) using linear interpolation, and center-cropped to 144 × 144 × 18 voxels. T2W and diffusion-weighted images were *z*-score-normalized to an intensity distribution with zero mean and unit standard deviation [11, 13]. The ADC intensities were divided by 3000 to retain their clinically relevant numerical significance, and to allow the model to quantitatively inspect ADC values, comparable to clinical routine [11, 14].

Lesions were scored by experienced uroradiologists (> 8 years’ experience) according to the Prostate Imaging – Reporting and Data System (PIRADS) version 2 guidelines [13]. All radiologist-identified lesions were manually delineated on each slice using the T2W and ADC scans. Segmentation was performed by the uroradiologists, or by research fellows under their supervision. For both tasks, the radiologists were blinded to all clinical information including pathological results. Clinical parameters including age, PSA, and PSA density (PSAd) were also collected for all prior and current exams.

### MRI surveillance features

MRI surveillance is increasingly used, but the number of sequential imaging examinations is currently too low (even when including data from different centers) to develop a deep learning system that directly compares two images. A hybrid approach that uses DL on single–time point MRI with a feature-level combination may be a potential solution. DL models for the detection of csPCa in MRI are usually trained to directly reproduce csPCa or PIRADS ≥ 4 annotations. For surveillance, however, the goal is to track also non-csPCa lesions that may later develop into csPCa. Therefore, our proposed solution uses a more sensitive DL detection algorithm trained to detect all radiologically detected lesions, to allow a machine learning classification layer to track the development of any detected lesions that could potentially develop into csPCa across two examinations.

**Fig. 2:**
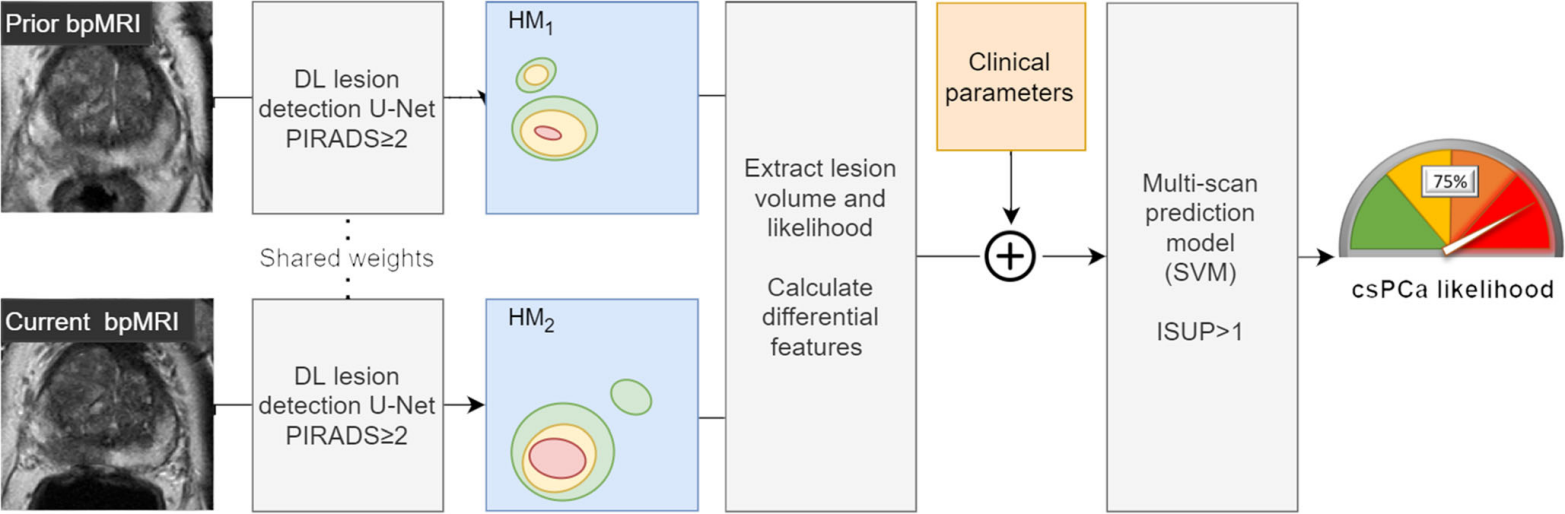
A visualization of the pipeline used to predict the likelihood of csPCa using sequential data (*D*_seq_). First, the two scans of a single patient are separately processed by the detection U-Net to generate two independent heatmaps for detected lesions (HM1, HM2). The U-Net was previously trained using dataset *D*_det_. Features for the likelihood and volume are then extracted from each of the heatmaps and used by the multi-scan classifier to predict the likelihood of csPCa at the time of the current examination

In more detail, a convolutional neural network (Fig. 2) was trained to detect all radiologist-identified lesions in any given scan. This includes low-risk lesions (PIRADS ≥ 2), conditioning the model to detect and track all radiologist-identified lesions. When no PIRADS ≥ 2 lesions were marked by the radiologist, the target lesion map was empty. The detection model was based on a 3D U-Net architecture and contained channel-wise squeeze-and-excitation modules and residual connections between consecutive convolutional blocks [15, 16]. Focal loss was used to counteract bias due to class imbalance in the dataset, and to implicitly calibrate the model at train-time [17–19]. This configuration has previously been shown to outperform similar models for the detection of PCa on bpMRI [20, 21].

The performance of the detection U-Net was monitored throughout training using a randomly selected validation set of 143 MRI scans (10% of dataset *D*_det_). The model with the lowest validation loss was selected as the final model. Images were augmented on the fly by applying random configurations of translation, rotation, mirroring, and elastic deformations [22]. The trained model was used to generate heatmaps for the prior bpMRI (HM_1_) and current bpMRI (HM_2_).

The heatmaps generated by the detection model were used to extract explainable volumetric and likelihood features for detected lesions in the prior and current bpMRI. Each patient’s prior scan was registered to the current scan using rigid registration on the T2W scans, to ensure that the features were extracted for the same region between prior and current scans. The total lesion volume was determined for the full scan at four different threshold values (0.1, 0.15, 0.2, and 0.25), by determining the number of voxels with a predicted likelihood score above each threshold. An overall likelihood score for each scan was determined by taking the maximum predicted likelihood in the corresponding heatmap. Differential features for the likelihood score, lesion volume, PSA, and PSAd were computed by calculating the differences between the prior and current exams.

A support vector machines (SVM) classifier with a linear kernel was trained to detect csPCa at the time of the current bpMRI using the features extracted from the images in *D*_seq_. This classifier was selected because of its effectiveness for feature-wise classification, and its simplicity in terms of hyperparameters [23]. PCa significance was evaluated on a per-examination level, as a histopathological grade of ISUP > 1 (Gleason Sum Score [GSS] > 6) at current biopsy (MRI-targeted or systematic).

### Experiments

Three diagnostic classifiers were created and compared: (1) a baseline classifier that only used the latest available MRI scan, (2) a surveillance classifier that also incorporated differential volumetric and likelihood scores, and (3) an extended surveillance classifier with access to clinical parameters. An overview of the features used for each classifier is shown in Supplementary Table 2.

### Statistical analysis

Receiver operator characteristic (ROC) curves were generated for each classifier using leave-one-out cross-validation. The classifiers were compared for statistical differences in diagnostic performance using DeLong’s test for paired ROC curves. An ROC curve was generated for the radiologist PIRADS score (determined with access to patients’ previous scans, but blinded to biopsy results and model predictions) for comparison. Sensitivity and specificity were determined based on Youden’s *J* statistic. Bootstrapping (*n* = 5000) was performed to generate 95% confidence intervals (CI) for each metric.

All statistical analyses were performed in R version 4.0.4 with the pROC package. The detection U-Net was implemented using TensorFlow 2.2.0. The SVM classifier was implemented in R using caret version 6.0-88.

## Results

An overview of the clinical characteristics at the prior exam is presented in Table 1. At the current evaluation, biopsy showed csPCa in 21 out of the current 78 examinations, while 57 showed non-csPCa. No significant differences were found in the baseline characteristics between groups with and without csPCa at the current examination.

**Table 1:** An overview of the baseline clinical characteristics (median, IQR) of the study cohort at the prior bpMRI. *p* values were calculated using the Mann-Whitney *U* test to evaluate differences between groups with and without csPCa at the current examination

Parameter	Total cohort (*n* = 78)	csPCa (*n* = 21)	Non-csPCa (*n* = 57)	*p* value
Age, years	71 (66.5–73.2)	70 (65.6–74.4)	71 (67.5–73)	0.7653
PSA, ng/mL	7.7 (6.2–10)	8.6 (7.4–11)	7.4 (6–10)	0.2943
PSA density	0.17 (0.12–0.23)	0.16 (0.13–0.22)	0.17 (0.11–0.23)	0.7439
Gland volume, cc	49.5 (32.25–57.75)	52.75 (30–56)	45 (33–60)	1.0
Follow-up, months	21 (14–32)	25 (15–30)	20 (13–32)	0.3857
Benign biopsy, *n*	45 (58%)	11 (24%)	34 (76%)	-
Gleason 3 + 3 = 6, *n*	33 (42%)	10 (30%)	23 (70%)	-

The DL detection model was trained for 48 h on a 32GB NVidia Tesla V100 GPU, for a total of 169 epochs.

**Fig. 3:**
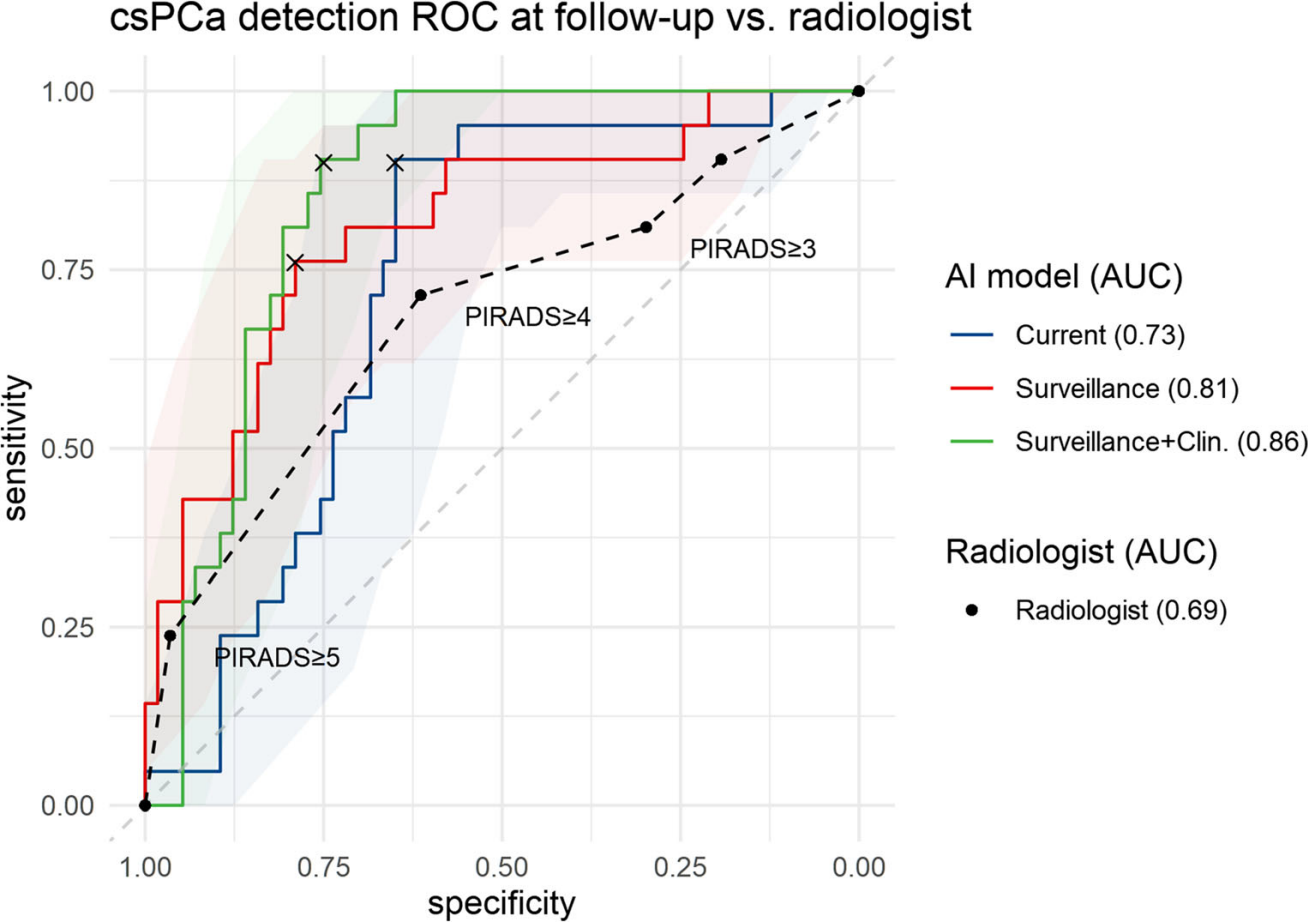
Examination-level ROC curves for the detection of csPCa at follow-up for each of the AI classifiers described in “Experiments.” Transparent areas behind each of the curves indicate bootstrapped 95% confidence intervals. The ROC for the radiologist assigned PIRADS scores is shown as the dotted line. Cross markers for the AI models indicate the sensitivity and specificity at the optimal cutoff point determined using Youden’s *J* statistic

The performance of each of the AI classifiers is presented in Fig. 3.

### Effect of including surveillance features

**Fig. 4:**
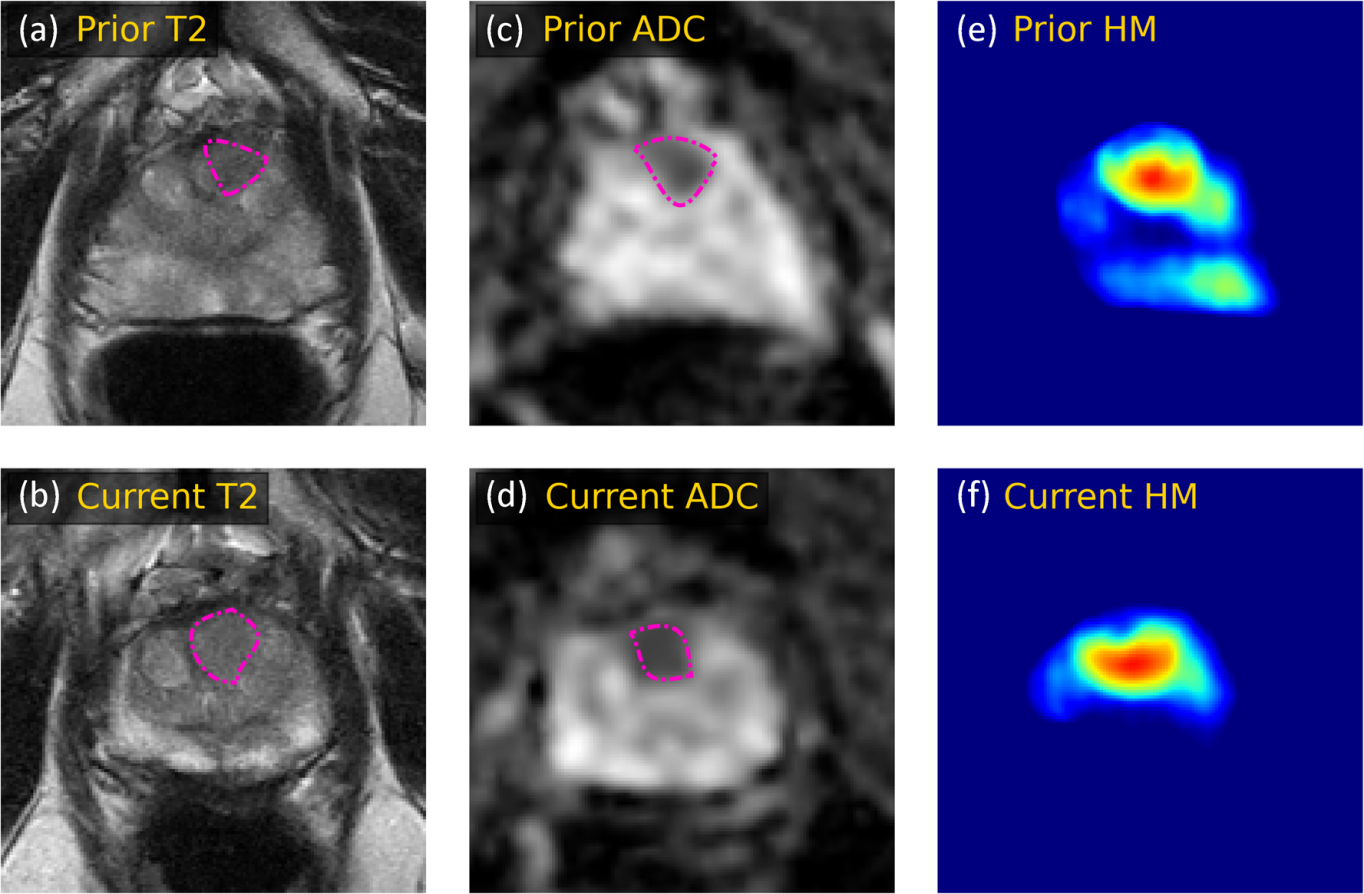
A 70-year-old man with a transitional zone lesion (delineated by the purple outline in each image) that was correctly diagnosed as non-csPCa by the surveillance AI. The initial biopsy at the prior scan was negative for PCa, and the targeted repeat biopsy at the current examination showed non-csPCa (Gleason 3 + 3 = 6). The figure shows T2-weighted images (**a**, **b**) and ADC maps (**c**, **d**), and corresponding detection heatmaps for the prior and current examination (**e**, **f**). The detection heatmap of the second examination showed a mostly unchanged volume and likelihood score. The radiologist assigned a score of PIRADS 4

**Fig. 5:**
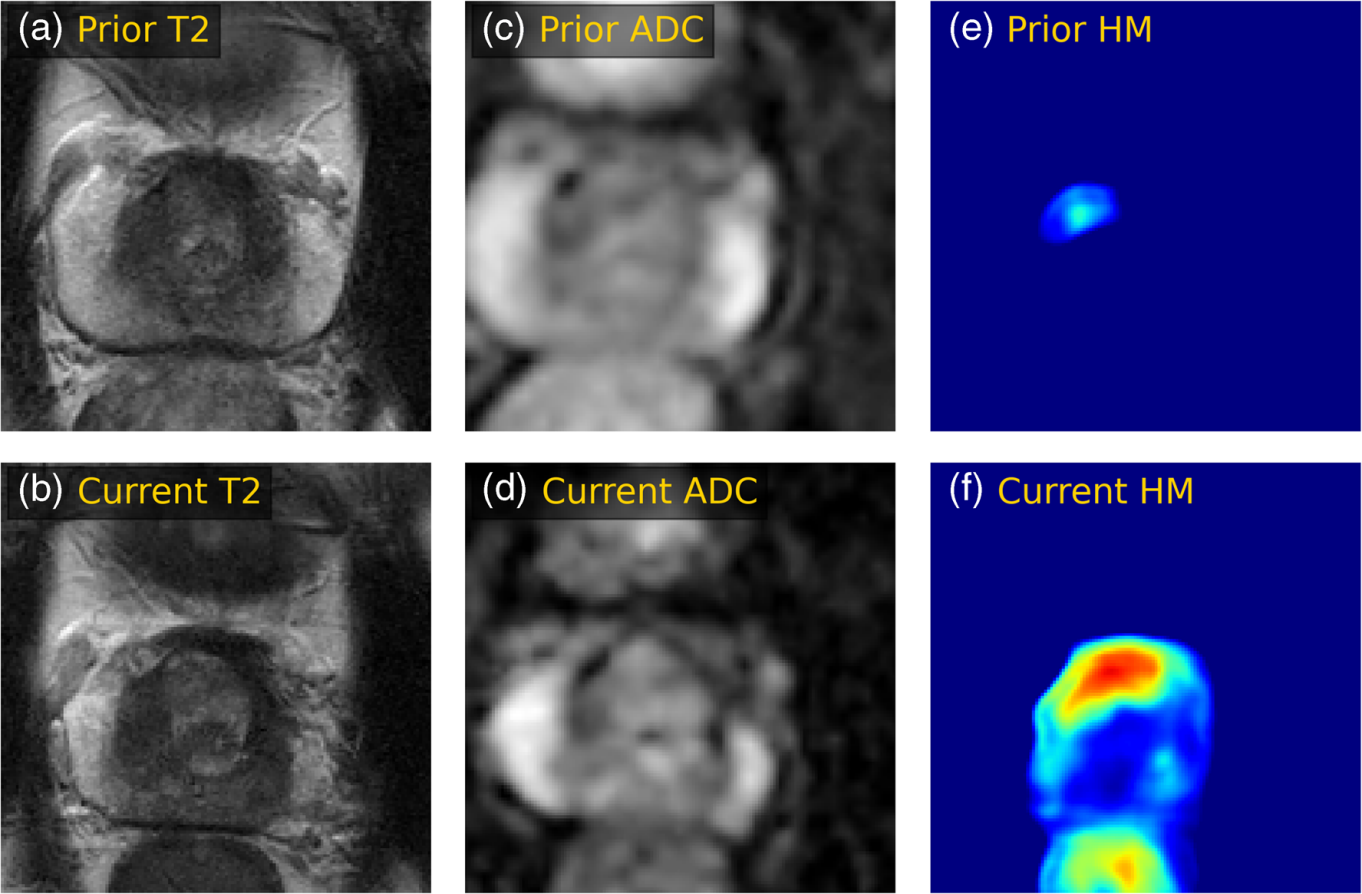
T2-weighted images (**a**, **b**) and ADC maps (**c**, **d**), and corresponding detection heatmaps (**e**, **f**) showing progression in a detected transitional zone lesion in a 66-year-old man on AS. The heatmaps show that a lesion was detected by the U-Net in both scans, and an increase in likelihood score and volume was recorded over a 4-year follow-up. The radiologist reported no PCa localization in the scan and assigned a score of PIRADS 1. A systematic repeat biopsy at the current examination revealed a Gleason score of 3 + 4 = 7. The surveillance AI correctly classified this patient as csPCa based on both scans

The classifier that only used the current scan reached an AUC of 0.73 (CI: 0.61, 0.84). The addition of sequential MRI data significantly (*p* = 0.04) improved the AUC to 0.81 (CI: 0.69, 0.91). The sensitivity and specificity of the latter model were 0.76 (CI: 0.57, 0.94) and 0.79 (CI: 0.68, 0.89), respectively. Figures 4 and 5 show example cases that were correctly classified using the surveillance classifier.

### Inclusion of clinical parameters

The inclusion of clinical parameters yielded an AUC of 0.86 (CI: 0.77, 0.93). Sensitivity and specificity were 0.90 (CI: 0.76, 1) and 0.75 (CI: 0.64, 0.86), respectively. The inclusion of clinical parameters did not result in a significantly improved AUC compared to the surveillance classifier without clinical parameters (*p* = 0.22).

### Comparison to radiologist performance

The AUC for the radiologist was 0.69 (CI 0.54, 0.81). Sensitivity and specificity were 0.71 (CI: 0.5, 0.9) and 0.61 (CI: 0.48, 0.74), respectively, when considering PIRADS ≥ 4 findings as the threshold for a positive csPCa detection. The surveillance classifier with access to clinical parameters achieved a significantly higher AUC than the radiologist (0.86 vs. 0.69, *p* = 0.02).

## Discussion

To our best knowledge, this is the first study to investigate a hybrid DL/machine learning system for automatic MRI surveillance in PCa. Our results show that our proposed surveillance AI was able to pick up explainable, diagnostically relevant changes in sequential MRI. The dual-scan surveillance AI achieved an AUC of 0.81, while the single-scan model was significantly less accurate (AUC 0.73). The results presented in this feasibility study provide a baseline for future explorations using AI for detecting lesion changes on MRI and correlation with clinical outcomes. By extending the surveillance model with clinical parameters including PSA, PSA density, and changes therein, the AUC was increased by 0.05. This suggests that sequential MRI information and clinical parameters provide complementary information for the diagnosis of csPCa and that these parameters can be effectively combined using a feature-level classification layer. In our experiments, the combination of clinical parameters and MRI surveillance enabled the system to accurately detect csPCa in cases that were not detected using sequential MRI features only. This is in line with Felker et al, who reported the combination of PSA density, lesion volume, and radiological changes between examinations as potentially useful to defer unnecessary repeat biopsies [7]. The inclusion of prior biopsy results could potentially further increase diagnostic performance; however, this was not explored in the current study because a combination of systematic and MRI-targeted biopsies was used for the prior study, which may have resulted in unstable predictions due to differences in sampling techniques.

Both AI surveillance systems (i.e., without and with clinical parameters) achieved a better diagnostic performance than the radiologist. This result can be explained by the consistency and precision in volumetric assessment and likelihood scoring by the DL detection model. To quantify progression, it is critical that the individual measurements for lesion volume and likelihood are determined consistently, both within and between patients. Current PRECISE guidelines for the evaluation of radiological changes do not clearly specify how to determine volume and likelihood scores from individual scans for comparison [6]. MRI surveillance of PCa can improve if AI provides more consistent and accurate scoring method to better compare scans. With surveillance AI, the diagnostic performance of serial MRI may become accurate enough to consistently exclude csPCa, to defer unnecessary biopsies in men on AS.

In this study, a U-Net was trained to sensitively detect all PCa in individual scans, including non-significant lesions. This approach is different from most other studies investigating computer-aided diagnosis of PCa, in which DL models are commonly trained to detect only csPCa, which results in a model that minimizes its response to non-csPCa lesions. This prevents accurate estimations of the volume and likelihood score of non-csPCa lesions that may develop into csPCa in subsequent examinations. Our results show that a more sensitive PCa detection model can be used to track also non-significant lesions, to obtain differential volume and likelihood features with diagnostic value for the detection of csPCa.

Only one other study, by Sushentsev et al (2021), investigated the use of AI for automatic detection of csPCa on sequential prostate MRI scans. They used changes in radiomics features for the similar task of detecting pathological grade progression in an AS cohort [24]. Their best performing AI model achieved an AUC of 0.82, which is similar to the performance achieved in the present study. However, their approach relied on manual segmentations provided by experts for the extraction of radiomic features in both scans, while our proposed system leverages DL to achieve a similar performance without any need for expert input. Moreover, their study used data from a single institution, whereas our analysis included patients from multiple centers.

Our study has several limitations. Firstly, the dataset used to train and validate the sequential classifier was relatively small. Although our results show the feasibility of sequential MRI data to improve the detection of csPCa, the availability of a larger dataset is required to further validate the proposed approach. With larger datasets, more powerful classifiers could be explored that require optimization of hyperparameters. Secondly, some bias may have been introduced to the detection model by not including patients with multiple scans in the training data for the detection U-Net (*D*_det_). This decision was motivated by the limited number of sequential MRI data, and the already large size of *D*_det_. Thirdly, the mixture of 3 T and 1.5 T scanners in our dataset may have affected the deep learning output. However, our detection model was also trained using a combination of 1.5-T and 3-T scanners and should therefore be conditioned to perform well on both. Furthermore, all models (and the radiologists) were evaluated on the same scans; therefore, the comparative performance should be representative. Lastly, there was a substantial variation in the time between the prior and current examination. However, this is comparable to similar studies and can be explained by the lack of guidelines regarding follow-up timing [7, 25].

In conclusion, our proposed AI-assisted surveillance of prostate MRI can pick up explainable, diagnostically relevant changes with promising diagnostic accuracy.

## Supplementary information

ESM 1 (DOCX 36 kb)

## Abbreviations

3D: Three-dimensional
ADC: Apparent diffusion coefficient
AI: Artificial intelligence
AS: Active surveillance
AUC: Area under the curve
bpMRI: Biparametric magnetic resonance imaging
CI: Confidence interval
csPCa: Clinically significant prostate cancer
DL: Deep learning
DWI: Diffusion-weighted imaging
GSS: Gleason Sum Score
ISUP: International Society of Urological Pathology Grade Group
MRI: Magnetic resonance imaging
PCa: Prostate cancer
PIRADS: Prostate Imaging Reporting and Data System
PRECISE: Prostate Cancer Radiological Estimation of Change in Sequential Evaluation
PSA: Prostate-specific antigen
PSAd: Prostate-specific antigen density
ROC: Receiver operating characteristic
SVM: Support vector machines
T2W: T2-weighted imaging

## Declarations

### Guarantor

The scientific guarantor of this publication is C. Roest.

### Statistics and biometry

No complex statistical methods were necessary for this paper.

### Methodology

• retrospective

• experimental

• multicenter study
