# Supplementary material for: AI-assisted biparametric MRI surveillance of prostate cancer: feasibility study
Source: Eur Radiol. 2022 Aug 12;33(1):89–96. doi: 10.1007/s00330-022-09032-7 (PMC9755080; doi:10.1007/s00330-022-09032-7)
Supplement: Supplementary file 1 — (DOCX 36 kb) [file 330_2022_9032_MOESM1_ESM.docx]

### Supplementary data 1

Supplementary Table 1. Overview of the care institutions included in the dataset. Hospitals A and C are tertiary care academic hospitals.

| Name | Vendor | Type of MRI system | Patients |
| --- | --- | --- | --- |
| Hospital A | Siemens | Skyra 3T, Prisma 3T, Aera 1.5T | 123 |
| Hospital B | Philips | Ingenia 3T, Achieva 1.5T, Intera 1.5T | 349 |
| Hospital C | Siemens | Skyra 3T, Prisma 3T | 861 |
| Hospital D | Philips | Achieva 1.5T | 89 |
| Hospital E | Philips | Ingenia 3T, Intera 1.5T | 35 |
| Hospital F | Siemens | Avanto 1.5T | 25 |
| Hospital G | Siemens | Avanto 1.5T, Aera 1.5 | 12 |
| Hospital H | Philips | Ingenia 3T | 7 |
| Hospital I | Philips | Achieva 1.5T | 4 |
| Hospital J | Siemens | Achieva 1.5T, Avanto 1.5T | 4 |
| Hospital K | Siemens | Espree 1.5T | 3 |
| Hospital L | Philips | Achieva 1.5T | 1 |

### Supplementary data 2

Supplementary Table 2. An overview of the features that were used by the classification layers to detect csPCa at the current examination. Current only, Surveillance, and Surveillance + Clinical refer to classifiers created for the experiments described in Section 2.3. Change features refer to differences in features between prior and current examination.

| Variable | Current only | Surveillance | Surveillance + Clinical |
| --- | --- | --- | --- |
| Current lesion likelihood | ✓ | ✓ | ✓ |
| Current lesion volume at cutoffs ≥ {0.1, 0.15, 0.2, 0.25} | ✓ | ✓ | ✓ |
| Lesion likelihood change |  | ✓ | ✓ |
| Lesion volume change at cutoffs ≥ {0.1, 0.15, 0.2, 0.25} |  | ✓ | ✓ |
| Current PSA, PSA density, age |  |  | ✓ |
| Change in PSA and PSA density |  |  | ✓ |

### Supplementary data 3

Supplementary Table 3. MRI sequence parameters.

| **Sequence** | **T2-weighted imaging** | **Diffusion-weighted imaging** |
| --- | --- | --- |
| In-plane resolution range (mm) | 0.23 – 0.78 | 0.85 – 2.19 |
| Slice thickness range (mm) | 3 – 5 | 3 – 5 |
| Sequence orientation | Axial, sagittal, and coronal | Axial |
| Remarks | No endorectal coil | No endorectal coil  b-value of 800 s/mm^2^ used for calculated b-value of > 1400 s/mm^2^ and mono-exponentially calculated apparent diffusion coefficient map |
